# Inoculation hesitancy: an exploration of challenges in scaling inoculation theory

**DOI:** 10.1098/rsos.231711

**Published:** 2024-06-26

**Authors:** Alexandra Johnson, Jens Koed Madsen

**Affiliations:** ^1^ Department of Psychological and Behavioural Sciences, London School of Economics and Political Science Houghton Street, London WC2A 2AE, UK

**Keywords:** misinformation, disinformation, inoculation theory, better-than-average effect, trust

## Abstract

Inoculation theory research offers a promising psychological ‘vaccination’ against misinformation. But are people willing to take it? Expanding on the inoculation metaphor, we introduce the concept of ‘inoculation hesitancy’ as a framework for exploring reluctance to engage with misinformation interventions. Study 1 investigated whether individuals feel a need for misinformation inoculations. In a comparative self-evaluation, participants assessed their own experiences with misinformation and expectations of inoculation and compared them to those of the average person. Results exposed a better-than-average effect. While participants were concerned over the problem of misinformation, they estimated that they were less likely to be exposed to it and more skilful at detecting it than the average person. Their self-described likelihood of engaging with inoculation was moderate, and they believed other people would benefit more from being inoculated. In Study 2, participants evaluated their inclination to watch inoculation videos from sources varying in trustworthiness and political affiliation. Results suggest that participants are significantly less willing to accept inoculations from low-trust sources and less likely to accept inoculations from partisan sources that are antithetical to their own political beliefs. Overall, this research identifies motivational obstacles in reaching herd immunity with inoculation theory, guiding future development of inoculation interventions.

## 1. Introduction

Online misinformation is a significant societal problem [1]. Viral deceptions have become issues debated at length by governments, academics and concerned citizens alike. While there is reasonable disagreement on the scope of the problem [2], it seems clear that misinformation represents a societal challenge. It may inflame conspiracy theories that demonize outgroups, reinforce social identities and further polarization [3]. It may have affected the rise of authoritarian leaders throughout Europe [4], influenced the 6th January riot in the United States [5], and encouraged ill-informed self-treatments of COVID-19 [6]. Exposure to misinformation lowers compliance with public health guidelines and intentions to get vaccinated against common diseases [7], which may compromise herd immunity [8].

Given the potential damage of misinformation, several solutions have unsurprisingly been proposed to combat the problem: fact-checking, warning labels, detection algorithms, etc. However, many of these solutions can only be put in place after the misinformation has already gone viral and been caught by fact-checkers or social media administrators. In this article, we focus on inoculation theory as a method of combatting misinformation—specifically, on how to implement and scale the intervention. Inoculation theory posits that individuals can be fortified against persuasive messages by exposing them to weakened versions of the same messages, analogous to how vaccines work to make individuals resistant to a virus by exposing individuals to a weakened form of the same virus [9,10]. These ‘inoculations’ build psychological ‘antibodies’, enabling individuals to recognize and resist attempts to sway their beliefs or attitudes. By using this psychological vaccine to immunize individuals against misinformation, inoculation theory offers a promising approach to fostering a more resilient and discerning populace.

McGuire developed inoculation theory well before the rise of the internet, but his metaphor seems particularly apt as misinformation online spreads similarly to a virus. Persuasive information can spread quickly from person to person, replicating and evolving to ‘infect’ as many people as possible [11]. The natural solution to a rapidly spreading virus is a vaccine, and as such inoculation theory has been used to successfully build resistance against climate change misinformation [12,13], anti-vaccine misinformation [14] and political misinformation [15].

Gamified inoculations, such as the ‘Bad News’ game, are one of the most prominent inoculation methods. These gamified methods use a technique-based inoculation approach to combat misinformation, aiming to develop a broad-spectrum vaccine against online manipulation. Researchers used known manipulation tactics often employed in spreading misinformation (such as highly polarizing language) and created the game where players assume the role of a fake news producer and use the strategies firsthand to spread misinformation. The assumption is that people who play the game and use tactics often associated with misinformation will be better at spotting messages that contain these techniques. When ‘Bad News’ was tested on thousands of voluntary participants, they found that it significantly improved individuals’ ability to recognize manipulative techniques, irrespective of their political ideology, age, gender or education [16,17]. Multiple successful active inoculation games, like Go Viral! [18], Harmony Square [7], Cranky Uncle [19] and Spot the Troll Quiz [20] have demonstrated that isolating and gamifying manipulative techniques, generally enhance people’s ability to recognize problematic content.

Recent research has found that good game design is essential for creating this effect. Games without feedback may primarily influence response bias, leading people to become more sceptical of both inaccurate *and* accurate information [21]. The effectiveness of inoculations also tends to decay over time unless regular ‘boosters’ are administered [22,23]. Both response bias and memory decay can be mitigated by making games that involve quizzes implementing the lessons learned, and offering formative feedback on those quizzes. These work to strengthen memory by applying the knowledge gained in-game, and cementing the differences between real and false news by offering corrections [24]. Another recent report pointed out that inoculation may make people more able to identify problematic content, but not improve their overall truth discernment without the help of accuracy prompts [25].

In the present work, we focus on another obstacle for inoculation theory: scalability. Because of its laboratory success in building resistance to misinformation, there is a growing interest in scaling up inoculation theory interventions to create ‘herd immunity’ [22]. However, most inoculation research published so far has used highly biased samples, specifically individuals who were compensated to fully engage with interventions. This presents a significant issue as the obtained effect sizes are likely skewed towards the upper limit of what can be anticipated. Actual effect sizes for light-touch behavioural interventions tend to be considerably lower in real-world situations [26]. Despite the experimental success of inoculation games, researchers at Cambridge’s social decision-making lab recognized that games require a considerable investment of time and effort, limiting their reach. In response to this challenge, researchers worked with Google to develop short videos that offer a more passive, technique-based form of inoculation. In a lab setting, these led to improved recognition of manipulation techniques, discernment between trustworthy and untrustworthy content, and better decision-making in content sharing [27]. To a lesser degree, these videos were also successful when deployed in the field as YouTube ads, improving recognition of manipulative techniques by 5–6%. Inoculation videos offer a convenient means of inoculation against misinformation. In the present research, they act as my hypothetical inoculation intervention in testing potential inoculation hesitancy. As a low-effort inoculation, any hesitancy to engage with this intervention is likely to be exacerbated in longer or more high-effort interventions.

Instead of testing the effectiveness of different inoculation methods, we ask whether people would be willing to engage with inoculation interventions to begin with. Sander van der Linden acknowledges the challenge of ‘psychological vaccine hesitancy’ in his book FOOLPROOF, stating, ‘Some people might not want the vaccine or the format in which you are offering it … what can we do for those people who might not be open to participating in our interventions?’ (p. 231) [28]. In this article, we ask whether people are interested in being inoculated, and if they see misinformation as a problem that *they* encounter or if they believe misinformation to be an issue that influences other people (Study 1). We further ask whether people are willing to engage with inoculation from sources they deem more or less trustworthy (Study 2), as this points to a central challenge in rolling out the inoculation against misinformation. It is plausible that more cynically minded people may not trust the motivations of researchers from elite universities, or the tech companies and governments they may partner with for funding. If reliability influences the willingness to take the inoculation in the first place, it may pose a barrier to rolling out the interventions. We expand on the motivation for Studies 1 and 2 in the following.

### 1.1. Extending the metaphor: inoculation hesitancy

Extending the metaphor that inoculation theory is based on reveals some of the potential challenges with achieving herd immunity. In recent years medical professionals have faced major hurdles in convincing the population to voluntarily get vaccines, regardless of their proven effectiveness. Vaccine hesitancy is defined as, ‘the delay in acceptance or refusal of vaccination despite the availability of vaccination services’ [29]. There are several identified causes of vaccine hesitancy, but the two major ones that can be applied to inoculation theory are (i) lack of need and (ii) lack of trust. We explore both in this article with regard to inoculation and misinformation. These factors were identified as part of a systematic analysis of vaccine hesitancy [30] and will be used in the present research as a framework for outlining the potential causes of inoculation hesitancy. We define inoculation hesitancy as the lack of motivation to engage with or the outright avoidance of inoculation interventions. This article explores whether Kumar *et al*.’s [30] demotivational drivers exist in the case of misinformation inoculations by conducting two online survey studies. These insights aim to aid in developing more engaging inoculation interventions.

In the case of vaccines, the vaccine-hesitant identify their own ‘lack of need’ as a major cause of reluctance. In Kumar *et al*.’s [30] review, they found that ‘some were of the view that it was unnecessary as they rarely contracted infectious diseases, the vaccine would be ineffective, or that their immune system was sufficient to handle the infection’ (p. 6). When people think they are personally immune to the disease, or they do not think the disease is a problem at all, they are unlikely to seek out a vaccine and may even try to avoid it. In fact, a meta-analysis has shown that the amount that people perceive themselves to be vulnerable to health problems is predictive of the likelihood that they will engage in health-promoting behaviours [31]. If individuals are genuinely unlikely to catch a disease, their disinterest in health measures is not a problem. However, when those who have ‘unrealistic optimism’ about their immunity choose to avoid learning about or adopting preventive measures because they don’t recognize any personal risk, it can have unnecessary consequences [32]. Indeed, a Bayesian model that includes subjective belief on contagion and risk to health shows that people who are hesitant to take vaccines for the common cold and COVID-19 score lower on these characteristics [33].

The existing body of literature on inoculation theory tends to overlook the voluntary nature of its real-world application, neglecting the pivotal role of individual motivation in accepting inoculation measures. Most people agree that misinformation is a problem, and a solution is needed, but on the individual level, it is unclear if people feel that the problem is something that affects them directly. One poll shows that 95% of Americans acknowledge that they view misinformation as a problem, but only 21% think they have personally shared misinformation [34]. This implies that in the case of misinformation, there may be perceived immunity where people believe that they are not affected by the problem in the same way as the ‘average’ person. People tend to rate their abilities and character traits as better than average. Although only half the population can be above average (or, at least, the median) on a given characteristic, most people believe that they are above average. This phenomenon, named the better-than-average effect, or sometimes the illusory superiority effect [35], has been documented across different dimensions. People rate everything from their intelligence [36] to their driving skills [37], more favorably than the average person’s abilities.

In the case of misinformation, this could result in those who are most in need of inoculation being the least likely to engage with inoculation interventions. When faced with evidence or input that challenges an individual’s perceived competence, they may dismiss or discount it, trusting their inflated self-assessment [32]. This dismissal could prevent them from recognizing the value of a psychological vaccine and hamper their willingness to engage in misinformation interventions. Study 1 explores whether Americans fall prey to the better-than-average effect when assessing their own capabilities in relation to misinformation and the need for inoculation.

Aside from believing that misinformation is something that happens to other people, lack of trust in the people and institutions behind vaccines may also play a role in vaccine hesitancy. This lack of trust caused difficulties in scaling up vaccination and reaching herd immunity, partially because of a lack of trust in the people and institutions distributing the vaccine, and partially because of the politicized nature of COVID-19 [30]. Over the years, researchers have emphasized the significant role of source trustworthiness in persuasion [38].

Yet, the discussion of inoculation theory generally seems to treat the inoculation as ‘sourceless’ while at the same time acknowledging that the source of the misinformation is key. The only research that has been done on the source of the inoculation has found that in general, the more positively a recipient perceives the source of the inoculation, the more effective the inoculation process tends to be [39,40]. However, this doesn’t acknowledge how the source plays into the decision of whether to inoculate yourself or not. Source credibility not only affects how people process information [41,42], but also how people select which information to consume [43]. While high-credibility sources do not necessarily get more exposure, low-credibility sources often deter engagement [44]. Because of the expensive nature of disseminating inoculation interventions, researchers sometimes must collaborate with governments [18] and tech companies [27], but there are broad swaths of the population that may be sceptical of the motivations of these collaborators and be deterred from participation.

Selectivity about the content we consume is not only driven by levels of trust but also by political attitudes. Misinformation has been heavily politicized with some viewing it as a problem perpetuated or even created by partisan adversaries [45]. Political elites and media personalities increasingly use terms like misinformation and fake news to discredit information they do not agree with and delegitimize political rivals [46,47]. Even the term ‘inoculation’ is political because of its association with vaccines. As such, there is considerable variation in support for misinformation interventions depending on partisanship. Saltz *et al*. [48] found that support for interventions such as social media labelling and downranking differs considerably by political party, trust in institutions and frequency of social media usage. Yet, even though widespread inoculation will require overcoming partisan feelings about misinformation interventions, very little research has been done on how the alignment of the ‘inoculator’ could influence engagement with interventions. The second study investigates how partisan sources and various levels of trustworthy sources affect inoculation uptake.

## 2. Material and methods: Study 1

Throughout both surveys, we referred to the inoculation intervention as a ‘series of misinformation training videos’ as we found this to be the simplest and most accessible way of describing the short technique-based misinformation inoculation videos developed by Roozenbeek *et al.* [27]. We avoided using the term inoculation to avoid complicating the perceptions of the participants with the possible association with vaccines. We used these videos as the theoretical inoculation intervention because they have proven effectiveness and offer the shortest time (2–3 min per video) to achieve inoculation which makes them the most convenient form of inoculation currently available. Participants in both surveys were recruited from the registered participant pool on Prolific, because it has been found to be higher quality than similar methods such as Amazon’s mTurk [49]. Compensation was provided to participants upon completion of the surveys through the Prolific platform. Both studies were pre-registered on Open Science Framework prior to any data being collected (Study 1: https://osf.io/byw7n/; Study 2: https://osf.io/df3r4).

### 2.1. Study 1: a comparative self-evaluation of misinformation perceptions

Study 1 explores the perception of a lack of need and whether individuals perceived themselves and their peers as vulnerable to misinformation and in need of inoculation interventions. This is the first aspect of vaccine hesitancy according to Kumar *et al*. [30]. We designed a survey using Qualtrics that compared perceptions of participants’ own experiences/capabilities to that of the average person regarding misinformation and their perceived need and willingness to partake in training to help spot misinformation. We used a comparative self-evaluation, and the questions were formatted in the indirect method, which has participants evaluate themselves and the average person on separate scales. By having participants rate themselves on separate scales instead of the direct method (which has participants evaluate themselves in comparison to the average person on one scale) or the forced choice method (where participants choose whether they rank above or below average). We were able to avoid some of the egocentrism and focalism that can typically skew tests of the better-than-average effect [49].

Participants were asked to evaluate five measures, both by looking at their own perceptions, and what they assumed to be the ‘average’ person’s perception. All questions were measured on a 7-point Likert scale. First, they were asked to estimate how frequently they and (in their view) other people ‘encounter information that they later find out is untrue or misleading’. Secondly, we ask how concerned they and an average person are about misinformation as a societal problem. Third, we asked them to rate how good they and an average person would be at detecting misinformation. We then described the five traits that Roozenbeek *et al*. [27] classify as prevalent misinformation techniques: emotional language, incoherence, false dichotomy, scapegoating and ad hominem attacks, and asked how good they and an average person would be at recognizing when a technique was being used to manipulate them. Lastly, we ask how much they and an average person would benefit from watching ‘training videos to make you more resistant to misinformation’ and how likely they and an average person would be to watch such training videos.

Demographic information was collected from the participants. This included questions about their political ideology, gender, age and education level. In addition to the conventional demographic questions, we included three questions measuring institutional trust: one about trust in the government, one about trust in educational institutions and one about trust in the media. These questions were added based on the observations of Saltz *et al*. [48] that trust in American public institutions robustly predicts support for all categories of misinformation interventions.

The survey was exploratory in nature, but in line with previous literature cited above, we expect the participants’ perceptions of themselves to be more positive than their perceptions of the average person. The comparison of self-perception and other-perception was conducted with a series of paired *t*-tests, and demographic effects were explored using one-way ANOVAs.

### 2.2. Participants

We used Prolific to recruit American adults (18+) who were native English speakers. The data were collected at the beginning of June 2023. An *a priori* power analysis was conducted with G* power to obtain 0.95 power to detect a medium effect size of 0.25 at the standard 0.05 alpha error probability. The minimal sample size required for detecting the main effect was approximately 142. A total of 157 participants were recruited through Prolific, but 6 were eliminated due to incomplete answers or failing to consent to the research, leaving a total of 151 participants. Participants were paid an equivalent of £9/h. The participants were split 49% female, 48% male and 3% unspecified gender. Politically they were more liberal, with 18% identifying as conservative, 23% as moderate and 59% as liberal (although 48% fell towards the middle between slightly liberal and slightly conservative). They were fairly well educated with 71% having at least an associate’s degree. The participants also skewed a bit younger with 63% under 45.

## 3. Results: Study 1

### 3.1. Results

**Figure 1.**
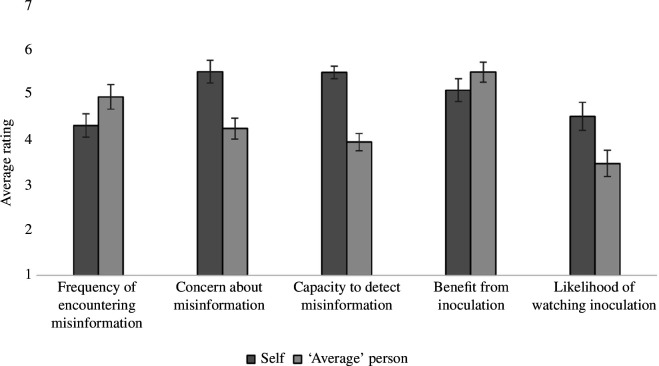
Comparative self-evaluation of misinformation and inoculation. *Note*. Each measure was rated on a 7-point Likert scale. *n* = 151. Error bars represent 95% confidence intervals.

In line with the pre-registered analysis plan (https://osf.io/6d5gr), we conducted a series of paired-sample *t*-tests across five different measures of self-comparison. We found a statistically significant difference in the rating of self versus the rating of the average person for all measures (see figure 1). The first measure tested was how often participants believed they were exposed to misinformation compared with how often they believed others were exposed to misinformation. Participants perceived their own exposure to misinformation (*M* = 4.32, s.d. = 1.63) as significantly lower compared with their perception of others’ exposure (*M* = 4.95, s.d. = 1.63), *t*(151) = −6.19, *p* < 0.001, *d* = 0.38. The second measure looked at how concerned participants were about misinformation and found that generally they rated their own concern (*M* = 5.51, s.d. = 1.51) as higher than the perceived concern of the ‘average’ person (*M* = 4.25, s.d. = 1.44), *t*(151) = 9.37, *p* < 0.001, *d* = 0.84.

The largest difference in the participants’ self-estimation compared with other-estimation was in the capacity to detect misinformation. To determine this score, we averaged the scores of the six questions about their own ability to detect misinformation and their perception of other’s ability to detect misinformation. The first question asked generally about how they perceived their ability/others misinformation detection abilities, and the following five questions outlined the specific techniques used by Roozenbeek *et al*. [20]. Consistent with the hypothesis, participants rated their own abilities as significantly better (*p*‐value < 0.001) than the average person across all questions. The overall capacity to detect scores shows that the participants evaluate their own ability (*M* = 5.49, s.d. = 0.83) to detect misinformation of any type as significantly better than the average person (*M* = 3.95, s.d. = 1.2), *t*(151) = 13.33, *p* < 0.001, *d* = 1.51.

The next measures focused on perceptions of inoculation interventions. The first tested whether participants felt they would benefit from watching short training videos designed to make them more resistant to misinformation. Participants did generally think they would benefit (*M* = 5.1, s.d. = 1.53), but they believed others (*M* = 5.5, s.d. = 1.38) would benefit more *t*(151) = −4.08, *p* < 0.001, *d* = 0.28. The final measure looked at the willingness of participants to watch short videos to make them more resistant to misinformation and how willing they believed the average person would be to watch those videos. We found that the overall average score fell in the middle of a 7-point Likert scale with a mean of 4.52, indicating between ‘neither likely nor unlikely’ and ‘slightly likely’ to voluntarily watch misinformation training videos. However, though it was somewhat low for the self-rating (*M* = 4.52, s.d. = 1.88), participants’ perception that others (*M* = 3.48, s.d. = 1.71) might voluntarily watch misinformation training videos was even lower, *t*(151) = 8.14, *p*<.001, *d* = 0.58.

As an exploratory measure, we tested the effect of demographic variables on the data through a series of one-way ANOVAs. The most consistent demographic difference was age. Table 1 shows the variations in means by age for the main measures. (A table of all demographic effects can be found in electronic supplementary materials.) The analysis shows a significant effect of age on the self-ascribed likelihood of watching inoculation videos, *F*_6,144_ = 4.39, *p* < 0.001. There was a similar variation of age in participants' perception of the likelihood of others watching inoculation videos, *F*_6,144_ = 5.7, *p* < 0.001. Post hoc comparisons using the Tukey HSD test indicated that the mean self-ascribed likelihood to watch score for those in the 35–44 age group (*M* = 3.59, s.d. = 1.83) was significantly lower than the score for the 45–54 group (*M* = 5.04, s.d. = 1.99), 55–64 group (*M* = 5.15, s.d. = 1.98) and 65–74 group (*M* = 6.17, s.d. = 0.83). The 65–74 age group score (*M* = 6.17, s.d. = 0.83) was also significantly higher than the score for those in the 25–34 age group (*M* = 4.33, s.d. = 1.68). The remaining scores showed no significant differences from each other. The pattern was the same for the perceived likelihood of others watching inoculation videos. Once again, those in the 35–44 age group rated others as significantly less likely to watch inoculation videos (*M* = 2.56, s.d. = 1.44) as compared with those in the 45–54 group (*M* = 4.09, s.d. = 1.59), 55–64 group (*M* = 4.05, s.d. = 1.88) and 65–74 group (*M* = 4.91, s.d. = 1.56). Once again, the 65–74 age group score (*M* = 4.91, s.d. = 1.56) was also significantly higher than the score for those in the 25–34 age group (*M* = 3.1, s.d. = 1.5). In general, these results suggest that age does influence the likelihood of engaging with inoculation interventions and that generally older participants (aged 45–74) had more faith that both themselves and others were more likely to watch inoculation videos, whereas younger participants (aged 25–44) had less faith in the likelihood that they or their peers would watch inoculation videos. Neither the youngest nor oldest age groups surveyed had any significant differences from other groups, though this is possibly because of their very small sample sizes (18–24, *n* = 9; 75–84, *n* = 1). Importantly, the better-than-average effect was still present across all ages, so regardless of age, participants believed they were more likely to watch a misinformation training video than the average person would be.

**Table 1.** Summary statistics results for age on main measures from Study 1.

age	freq self		freq other		benefit self		benefit other		watch self		*watch other*		*n*
	*M*	s.d.	*M*	s.d.	*M*	s.d.	*M*	s.d.	*M*	s.d.	*M*	s.d.	
18–24	4.56	1.67	5.00	1.22	4.89	1.76	4.89	1.62	3.89	1.76	3.44	1.67	9
25–34	4.50	1.73	5.50	1.56	5.04	1.48	5.65	1.31	4.33	1.68	3.17	1.50	54
35–44	4.28	1.46	4.81	1.23	4.47	1.63	4.88	1.36	3.59	1.83	2.56	1.44	32
45–54	4.30	1.64	4.74	1.68	5.09	1.44	5.70	1.26	5.04	1.99	4.09	1.59	23
55–64	4.10	1.77	4.10	2.07	5.65	1.35	5.80	1.40	5.15	1.98	4.05	1.88	20
65–74	3.75	1.48	4.50	1.68	6.25	0.97	6.08	1.31	6.17	0.83	4.92	1.56	12
75–84	4.00	NA	4.00	NA	6	NA	6	NA	6	NA	7	NA	1

*Note*. The main measures were rated on a 7-point Likert scale. Freq self and Freq other refer to the frequency of exposure to misinformation, benefit self and benefit other indicate if they believed there would be benefit from watching misinformation training videos, watch self and watch other refer to the likelihood of watching misinformation training videos. Skill at detecting misinformation and concern about misinformation were not included in this table as they had no significant variance by age. *** Indicates *p* < 0.001; ** indicates *p* < 0.01; * indicates *p* < 0.05.

There were also significant effects of age on the perceived frequency of others misinformation exposure, *F*_6,144_ = 2.36, *p* = 0.0334. Post hoc comparisons using the Tukey HSD test indicated that the mean frequency of other exposure score for those in the 55–64 age group (*M* = 4.1, s.d. = 2.07) was significantly lower than the score for the 25–34 group (*M* = 5.5, s.d. = 1.56). Perceptions of self-ascribed benefit of inoculation videos also varied significantly by age, *F*_6,144_ = 2.76, *p* = 0.0142. Post hoc comparisons using the Tukey HSD test indicated that the mean self-benefit score for those in the 65–74 age group (*M* = 6.25, s.d. = 0.97) was significantly higher than the score for the 35–44 group (*M* = 4.47, s.d. = 1.63). Indicating that the older age group thought they would benefit more from inoculation videos than those in the younger age group. Finally, perceptions of how much others would benefit from inoculation videos varied significantly by age, *F*_6,144_ = 2.22, *p* = 0.0441. Post hoc comparisons using the Tukey HSD test found that there were no individual pairwise comparisons that were significant, likely due to the weakly significant global effect.

Study 1 shows that participants believe they are less likely to encounter misinformation than the average person, but they are better able to detect it when they do. They believe that they are more concerned about misinformation than the average person and that they are more likely to watch training videos than the average person even if the average person would benefit more than they would. In line with predictions, these findings support the hypothesis that people exhibit an illusory superiority effect [26] when it comes to the topic of misinformation.

### 3.2. The importance of institutional trust

A pattern emerged in the ANOVAs measuring the variance in the likelihood of watching across different types of institutional trust. We found significant variance explained by institutional trust in the likelihood of watching scores. Specifically, there was a statistically significant difference in the self-ascribed likelihood of watching inoculation videos among the levels of government trust, *F*_6,144_ = 2.99, *p* = 0.00873. Post hoc comparisons using the Tukey HSD test indicated those who found the government moderately trustworthy in the first study (*M* = 5.53, s.d. = 1.37) were significantly more likely to say they would watch inoculation videos than those who found the government extremely untrustworthy (*M* = 3.57, s.d. = 2.12) and moderately untrustworthy (*M* = 3.86, s.d. = 1.9). This implies that lower government trust is associated with being less willing to watch inoculation videos. The trend was similar for significant variations in media trust, *F*_6,203_ = 2.94, *p* = 0.00973 and educational trust, *F*_6,203_ = 2.89, *p* = 0.0109.

When translating the Likert-type trust rating into ordinal variables, ordinal linear regressions corroborate the findings. Government trust correlates positively with the likelihood of watching inoculation videos (z-value = 3.96, *p* < 0.001), as does educational trust (*z*-value = 3.74, *p* < 0.001) and media trust (*z*-value = 3.47, *p* < 0.001).

## 4. Material and methods: Study 2

### 4.1. Study 2: the impact of source on the willingness to inoculate

Study 1 explored the perception of the scope of the problem. In Study 2, we explore the other facet of Kumar *et al.*’s [21] barriers to vaccine uptake, namely, the impact of trust. Specifically, we are interested in whether participants would engage with training videos against misinformation if they believe the source to be untrustworthy, as this would post a practical challenge for scaling up inoculation interventions for citizens who do not trust interventions developed at elite universities (e.g. The Bad News Game was developed at the University of Cambridge).

To test how the source of the inoculation changes the willingness to participate in inoculation interventions we conducted a pilot study (*n* = 47) where participants evaluated 17 possible sources on their trustworthiness and effectiveness with regard to misinformation inoculation. The purpose was to identify a high-trust source, a medium-trust source, a low-trust source and two partisan sources that could be used for the study (we pilot to avoid the trap that we choose sources *we* believe to be expert and trustworthy). As we conducted Study 2 with American citizens, the piloted participants were also from the United States. All sources were rated on a sliding scale of 1–100. Interestingly, all 17 sources were rated below 65 (out of 100), suggesting that no single source could be considered consistently high. However, the highest-rated source was an ‘Ivy League University’ rated at 62.3, the median rated source was ‘Meta’ (29.53) and the lowest-rated source was ‘The Russian Government’ at 11.89. In order to include political parties in the study, we tested ‘The Republican Party’ (23.21) and ‘The Democratic Party’ (32.34) as opposing partisan sources. We use these five sources in the study.

The study measured how likely people believe they are to voluntarily watch videos that inoculate them against misinformation and how that likelihood is affected by whether the video comes from a high-trust source, a low-trust source, a medium-trust source or a partisan source. Given the reviewed literature on the importance of trust and reliability in information search and belief revision as well as partisan observations in politics, we hypothesize the following:

*H1*: Participants are less likely to voluntarily watch inoculation videos if the source is less trustworthy.
*H2:* Participants are less likely to voluntarily watch inoculation videos if the source represents an opposite political affiliation compared to the participant.

In the survey, we used a within-subjects design and asked participants to reflect on two questions for five different trust scenarios. We ask participants to imagine that a team of researchers has developed a series of training videos. The team that has developed the videos claim they are designed to make viewers more resistant to the techniques used to spread misinformation. This description is in line with inoculation theorists’ description of the intention of these interventions and videos. For each scenario, participants are asked to imagine the team of researchers is from a different group, Harvard University (high-trust), Meta (medium-trust), the Russian government (low-trust), the Democratic Party (partisan trust left) and the Republican Party (partisan trust right). The order in which these scenarios appeared was randomized for each participant. For each scenario, they were asked (i) whether they would be likely to voluntarily watch the videos on a 7-point Likert scale ranging from ‘Extremely unlikely’ to ‘Extremely likely’; and (ii) whether they believe that if they were to watch the videos, they would get better at detecting misinformation as a result on a 7-point Likert scale ranging from ‘Definitely not’ to ‘Definitely yes’. The second question operates primarily to clarify the motivation of the answer to the first question. For instance, someone might want to watch a training video from a source out of curiosity even if they don’t believe it will benefit them, or they may believe a training video would benefit them but are unlikely to watch it out of laziness. By asking both questions, we can obtain more precise data about the effect of the source. We also ask the same series of demographic questions as Survey 1, with the addition of a question about how you are planning to vote in the next election to determine their political allegiance.

The data were analysed using independent *t*-tests comparing each source to the baseline likelihood to watch score established in the first survey. Whether political affiliation effects willingness to take an inoculation from a partisan source will be evaluated using a one-way ANOVA.

### 4.2. Participants

As in Study 1, we used Prolific to recruit American adults (18+) who were native English speakers. An *a priori* power analysis was conducted with G* power to obtain 0.95 power to detect a medium effect size of 0.25 at the standard 0.05 alpha error probability. The minimal sample size required for detecting the main effect was calculated to be approximately 210. During the data collection in late June 2023, four participants were excluded from the original sample size of 214 due to incomplete results or failure to give consent, yielding a total of 210 participants. Participants were paid an equivalent of £9/h. The participants were split 48% female, 51% male and 1% gender non-conforming. Politically they were more liberal, with 23% planning to vote Republican, 59% planning to vote democrat and 17% not planning to vote or voting Third Party. They were fairly well educated with 70% having at least an associate’s degree and skewed a bit younger with 68% under 45.

## 5. Results: Study 2

### 5.1. Results

**Figure 2.**
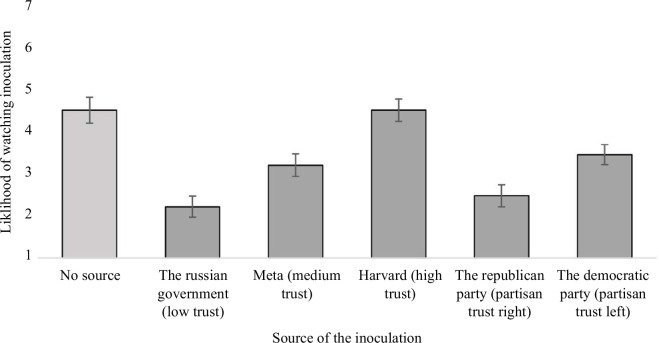
Source trustworthiness by likelihood of watching misinformation Inoculation videos. *Note*. The results of the no-source inoculation are from Study 1 (*n* = 151) whereas the rest of the results are from Study 2 (*n* = 210). All likelihood ratings were built on a 7-point Likert scale ranging from ‘Extremely Unlikely’ to ‘Extremely Likely’. Error bars represent 95% confidence intervals.

We hypothesized that people would be less willing to watch misinformation training videos from low-trust sources. In line with the pre-registered analysis (https://osf.io/df3r4), we ran a series of independent *t*-tests using the self-evaluated ‘likelihood of watching’ data from the first study (*n* = 151) as a baseline since it did not directly include a source and compared it to the willingness to watch data from each of the five sources. Participants were significantly less likely to say they would voluntarily watch both partisan sources, the low-trust and the medium-trust sources than the no-source condition, and the high-trust source was rated about the same as the no-source condition (see figure 2).

The participants rated their likelihood of watching inoculation training videos of the low-trust source (*M* = 2.22, s.d. = 1.8) significantly lower than the no-source condition (*M* = 4.52, s.d. = 1.88) meaning that participants are significantly less likely to say they would watch inoculation training videos from the Russian government, *t*(359) = 11.77, *p* < 0.001, *d* = 1.26. Meta, the medium-trust source (*M* = 3.21, s.d. = 1.93) was also rated significantly lower than the no-source condition, *t*(359) = 6.45, *p*  < 0.001, *d* = 0.68. The partisan left source (*M* = 3.46, s.d. = 1.89) and the partisan right source (*M* = 2.48, s.d. = 1.74) were both rated significantly lower than the no-source condition, *t*(359) = 5.29, *p*  < 0.001, *t*(359) = 10.64, *p*  < 0.001. However, the effect size, as measured by Cohen’s *d*, was *d* = 0.56 for the partisan left source, indicating a medium effect, but it was *d* = 1.14 for the partisan right source, indicating a large effect size. The only condition that was not significantly lower than the no-source condition was the high-trust condition (*M* = 4.52, s.d. = 1.96) which was not significantly different from the no-source condition at all, *t*(359) = −0.003, *p* = 0.9976. This supports the hypothesis that people are less willing to watch videos from sources they perceive as low in trust.

We further conducted *t*-tests to discover whether participants said that they were likely to benefit from the inoculation even if they weren’t as likely to say they were willing to watch the inoculation. Apart from the partisan sources, the participants rated their likelihood of benefitting from watching inoculation videos as significantly higher than their likelihood of voluntarily watching those videos. Even for the low-trust source, participants rated their likelihood of watching inoculation videos from the Russian Government (*M* = 2.22, s.d. = 1.8) as significantly lower than their likelihood of benefitting from the same videos (*M* = 2.45, s.d. = 1.6) at the *p* < 0.05 level, *t*(209) = −2.4, *p* = 0.01712. The difference was even more pronounced for the medium-trust source, where the likelihood of watching (*M* = 3.21, s.d. = 1.93) was lower than the likelihood of benefitting (*M* = 3.59, s.d. = 1.21), *t*(209) = −4.23, *p* < 0.001. The same pattern continued with the high-trust source, with the likelihood of watching inoculations from Harvard (*M* = 4.52, s.d. = 1.96) rated lower than the likelihood of benefitting from those videos (*M* = 4.82, s.d. = 1.55), *t*(209) = −3.36, *p* < 0.001. The benefit question was specifically added to evaluate whether lack of interest or laziness may factor into the likelihood of watching inoculation videos. This pattern of consistently seeing potential benefit from inoculation videos but still rating your likelihood of watching those videos as lower, implies that a certain level of apathy may affect the outcome of these results as well as the trustworthiness of the source. For the two partisan sources, participants also rated their likelihood of watching the videos as lower than their likelihood of benefitting from those videos, but there was not a statistically significant difference.

### 5.2. Inoculation across party lines

Aside from trust, we test whether people’s political affiliation influences their willingness to engage with training videos against inoculation. Like perceived trust, this is important for identifying potential practical barriers to rolling out interventions if they are seen as politically biased or coming from low-trust sources. To test this, one-way ANOVA tests were conducted to compare the effect of political party preference on willingness to watch partisan-sourced inoculation videos. This divides participants into their own political party preferences (Democratic Party, Republican Party, Third-Party or not voting). We hypothesized that people would be less willing to engage with training material from non-preferred political entities. This hypothesis was supported as there was a significant effect of political party preference on willingness to watch across party lines for both parties (see table 2).

**Table 2.** Willingness to watch partisan sources by voting preference.

voting preference	likelihood of watching Democratic Party inoculation		likelihood of watching Republican Party inoculation		
	*M*	s.d.	*M*	s.d.	*N*
Democratic Party	4.15	1.71	2.14	1.65	125
Republican Party	1.9	1.42	3.04	1.72	48
Third-Party	3.94	1.78	3.71	2.08	17
not voting	2.45	1.47	2.25	1.25	19

*Note*. The likelihood of watching each partisan source was rated on a 7-point Likert scale.

There was a significant effect of voting preferences on willingness to watch inoculation videos from the Democratic Party, *F*(3, 206) = 24.74, *p* < 0.001. Post hoc comparisons using the Tukey HSD test indicated that the mean likelihood of watching score for those intending to vote for the Democratic Party (*M* = 4.15, s.d. = 1.71) was significantly higher than the score for those intending to vote for the Republican Party (*M* = 1.9, s.d. = 1.42) and higher than those not intending to vote in the next election (*M* = 2.45, s.d. = 1.47). The willingness to watch score for those intending to vote Republican was significantly lower than those intending to vote Third-Party (*M* = 3.94, s.d. = 1.78). However, the Democratic voters did not differ significantly in willingness to watch inoculation videos from the Third-Party voters, nor did the Republican voters differ significantly from the non-voters.

On the opposite side of the political spectrum, the pattern held true in reverse. There was also a significant effect on the likelihood of watching inoculation videos from the Republican Party at the *p* < 0.001 level for the four conditions [*F*_3, 206_ = 6.66, *p* < 0.001]. Post hoc comparisons using the Tukey HSD test indicated that the mean likelihood to watch score for those intending to vote for the Democratic Party (*M* = 2.14, s.d. = 1.65) was significantly lower than the score for those intending to vote for the Republican Party (*M* = 3.04, s.d. = 1.73) and lower than Third-Party voters (*M* = 3.71, s.d. = 2.08). However, the Democratic voters did not differ significantly in willingness to watch inoculation videos from the non-voters, nor did the Republican voters differ significantly from the Third-Party voters and only slightly differed from the non-voters (*p*-adj = 0.044). In general, these results suggest that voters are unlikely to take inoculation from across party lines and non-voters are unlikely to take inoculation from either major political party.

Voting preferences did not affect the likelihood of watching inoculation videos from the Russian government or Meta, but they did affect the likelihood of watching inoculation videos from Harvard, *F*_3,206_ = 6.21, *p* < 0.001. Post hoc comparisons using the Tukey HSD test indicated that the mean likelihood of watching score for those intending to vote for the Democratic Party (*M* = 4.93, s.d. = 1.78) was significantly higher than the score for those intending to vote for the Republican Party (*M* = 3.6, s.d. = 2.09). However, there were no other significant differences within voting preferences.

### 5.3. The importance of institutional trust

**Figure 3.**
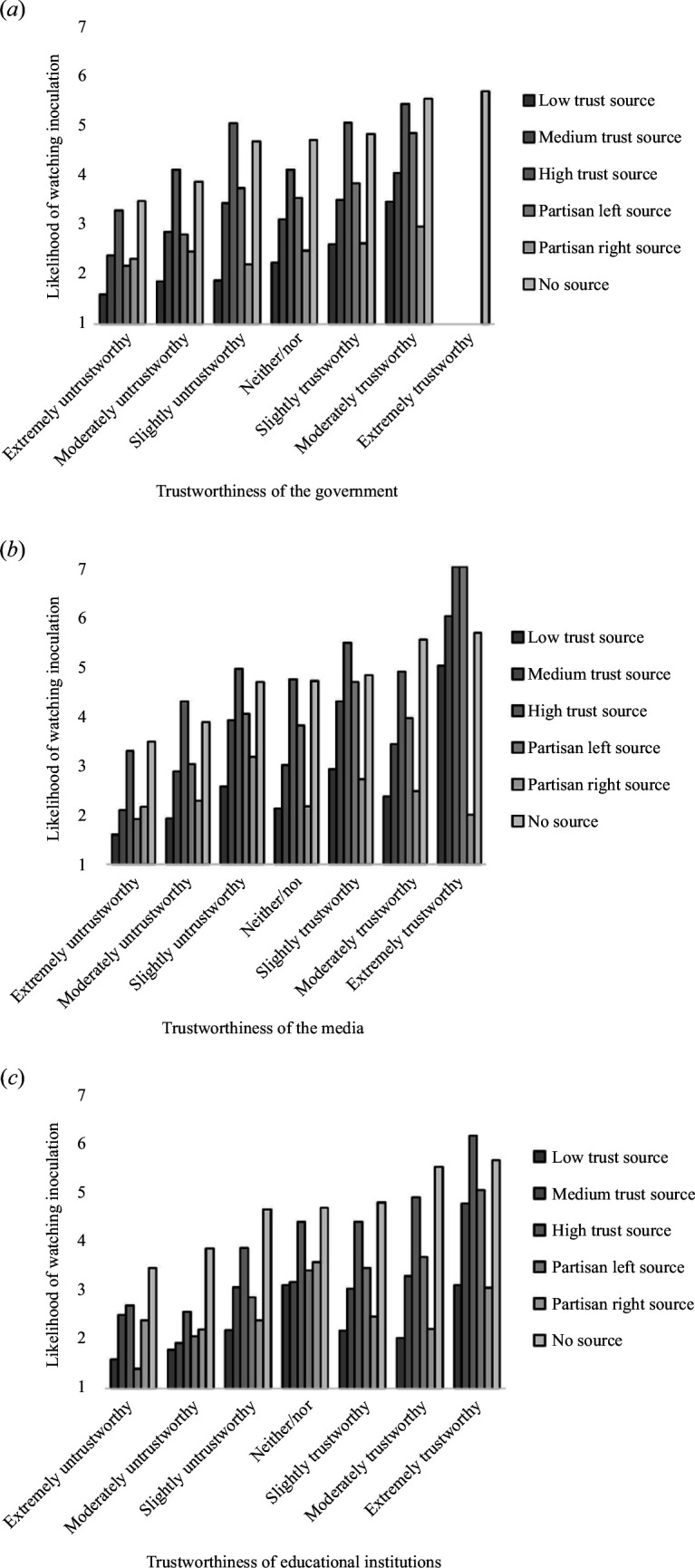
The effect of Institutional trust on likelihood to watch Inoculation videos. (*a*) Trustworthiness of the goverment; (*b*) Trustworthiness of the media; (*c*) Trustworthiness of educational institutions. *Note*. The no-source condition was derived from Study 1 (*n* = 151) while the rest of the conditions were derived from Study 2 (*n* = 210). In Study 2, zero participants rated the government as extremely trustworthy, hence the gap in the data.

We conducted a series of one-way ANOVAs on institutional trust for the five source conditions in the second study. We find statistically significant variance for every source condition except for the partisan right source (for all types of institutional trust) and the low-trust source (for educational trust only). Looking at the comparative bar chart (figure 3a,*b*,*c*), results show that the likelihood to watch score is generally higher when the institutional trust level is higher. This suggests that people’s belief in the trustworthiness of institutions moderates their willingness to engage with misinformation training videos across conditions.

There was a statistically significant difference in the likelihood of watching the high-trust source inoculation among the levels of government trust, *F*_5,204_ = 5.78, *p* < 0.001. Post hoc comparisons using the Tukey HSD test for the high-trust source likelihood to watch scores indicated that those who rated the government as extremely untrustworthy were significantly less likely to watch inoculation videos from the Democratic Party (*M* = 3.28, s.d. = 2.09) than those who rated the government as only slightly untrustworthy (*M* = 5.03, s.d. = 1.44), slightly trustworthy (*M* = 5.04, s.d. = 1.76) and moderately trustworthy (*M* = 4.83, s.d. = 1.93). The trend was similar for significant variations in media trust, *F*_6,203_ = 5.83, *p* < 0.001 and educational trust, *F*_5,204_ = 8.13, *p* < 0.001.

There was also statistically significant difference in the likelihood of watching the medium-trust source inoculation among the levels of government trust, *F*_5,204_ = 2.66, *p* = 0.0236. Post hoc comparisons using the Tukey HSD test for the medium-trust source likelihood to watch scores indicated that those who rated the government as extremely untrustworthy were significantly less likely to watch inoculation videos from Meta (*M* = 2.38, s.d. = 1.78) than those who rated the government as moderately trustworthy (*M* = 4.04, s.d. = 2.21). The trend was similar for significant variations in media trust, *F*_6,203_ = 6.61, *p* < 0.001, and educational trust, *F*_6,203_ = 3.59, *p* = 0.00211.

There was even significant variance in the likelihood to watch scores of the low-trust source by government trust, *F*_5,204_ = 4.38, *p* < 0.001. Post hoc comparisons using the Tukey HSD test for the low-trust source likelihood to watch scores indicated that those who rated the government as moderately trustworthy were significantly more likely to watch inoculation videos from the Russian government (*M* = 3.46, s.d. = 2.36) than those who rated the government as slightly untrustworthy (*M* = 1.88, s.d. = 1.56), moderately untrustworthy (*M* = 1.85, s.d. = 1.46) and extremely untrustworthy (*M* = 1.59, s.d. = 1.32). The trend was similar for significant variations in media trust, *F*_6,203_ = 2.72, *p* = 0.0146, but there was no significant variance by educational trust levels.

The partisan trust sources were different, the left-leaning source had similar variation by all types of institutional trust levels, but the right-leaning source had no significant variation at all. There was a statistically significant difference in the likelihood of watching the partisan left trust source inoculation among the levels of government trust, *F*_5,204_ = 7.78, *p* < 0.001. Post hoc comparisons using the Tukey HSD test indicated that for the partisan left source likelihood to watch scores for those who found the government extremely untrustworthy (*M* = 2.17, s.d. = 1.69) were significantly lower than the score for those who rated the government as only slightly untrustworthy (*M* = 3.73, s.d. = 1.43), neither trustworthy nor untrustworthy (*M* = 3.53, s.d. = 1.72), slightly trustworthy (*M* = 3.83, s.d. = 1.89) and moderately trustworthy (*M* = 4.83, s.d. = 1.93). Those who found the government moderately untrustworthy (*M* = 2.8, s.d. = 1.86) also have a significantly lower likelihood of watching those who rated the government moderately trustworthy (*M* = 4.83, s.d. = 1.93). The trend was similar for significant variations in media trust, *F*_6,203_ = 12.03, *p* < 0.001 and educational trust, *F*_6,203_ = 6.78, *p* < 0.001. (Full results with post hoc comparisons can be found in electronic supplementary materials.)

We translated the Likert-type trust rating into ordinal variables and found that ordinal linear regressions corroborate the findings. We find a statistically significant positive correlation between institutional trust and the likelihood of watching inoculation videos for every source condition except for the partisan right source (for all types of institutional trust) and the low-trust source (for educational trust only).

For the high-trust source (Harvard University), media trust (*z*-value = 5.04, *p* < 0.001), government trust (*z*-value = 4.31, *p* < 0.001) and educational trust (*z*-value = 5.95, *p* < 0.001) all demonstrate positive correlations with the likelihood of watching inoculation videos. Similarly, for the medium-trust source (Meta), positive correlations are observed for media trust (*z*-value = 4.62, *p* < 0.001), government trust (*z*-value = 3.36, *p* < 0.001) and educational trust (*z*-value = 3.62, *p* < 0.001). Both media trust (*z*-value = 3.13, *p* = 0.001) and government trust (*z*-value = 4.33, *p* < 0.001) correlate positively with the likelihood of watching an inoculation video from the low-trust source (The Russian Government). However, there is no significant correlation between educational trust (*z*-value = 0.81, *p* = 0.415) and the likelihood of watching an inoculation from the low-trust source.

The left-leaning source had positive correlations with all types of institutional trust, but the right-leaning source had no significant correlation with institutional trust at all. The likelihood of watching inoculation videos from the partisan left source was positively associated with media trust (*z*-value = 6.87, *p* < 0.001), government trust (*z*-value = 5.62, *p* < 0.001) and educational trust (*z*-value = 5.17, *p* < 0.001). For the partisan right source, no significant correlations are observed between media trust (*z*-value = 1.14, *p* = 0.251), government trust (*z*-value = 1.47, *p* = 0.140), educational trust (*z*-value = −0.41, *p* = 0.683) and the likelihood of watching inoculation videos, highlighting the differentiated impact of institutional trust across partisan sources.

## 6. Discussion

The present research explored the scalability of inoculation theory and individuals' willingness to receive inoculations against misinformation. This is a departure from typical inoculation studies, which focus on the effect of inoculation, instead we explore potential barriers to the implementation of such interventions. We investigated the concept of inoculation hesitancy to understand the psychological factors that might prevent the uptake of inoculation interventions. These studies uncovered a perceived lack of need for inoculation among individuals and a hesitance to engage with inoculations from sources that they don’t find trustworthy—demotivators that may be major obstacles in overcoming inoculation hesitancy. The results suggest that immunizing the public against misinformation may be an uphill battle.

In the first study, we examined the aspect of inoculation hesitancy related to the lack of need. The results demonstrated a clear pattern, participants rated their own capacity to detect misinformation significantly higher than they perceived the average person’s capacity, revealing that they may believe themselves to be less vulnerable to the effects of misinformation. This is in line with past studies of the better-than-average effect where people tend to believe in the superiority of their own abilities [50]. Additionally, individuals believed they were exposed to misinformation less frequently than other people. Yet, participants claimed to have a higher level of concern about misinformation compared with the average person, indicating their recognition of misinformation as a pertinent issue. These findings taken together imply that people view others as both the primary spreaders and victims of misinformation.

The first study also looked at the better-than-average effect in the context of potential inoculation interventions. The effect was consistent, with participants assuming the average person would benefit more from watching misinformation training videos than they would. This shows that they don’t believe improvement is as necessary for themselves as it is for others. Participants expressed only a moderate willingness to voluntarily watch misinformation training videos, possibly because of their confidence in their abilities to detect misinformation. Still, participants’ belief in their own superiority remained consistent, as they believed the average person would be even less likely to watch. This suggests that individuals tend to believe that they are less susceptible to misinformation and, therefore, might not perceive a pressing need for inoculation interventions for them personally.

We also explored demographic effects on misinformation and inoculation perceptions and found that age was a significant factor. Generally, younger age groups rated both themselves and others as less likely to watch misinformation training videos than their older counterparts. This finding has implications for any future campaigns intending to spread inoculation interventions. It suggests that the primary focus should be on younger age groups. However, it’s important to note that this pattern did not hold true in the second study, emphasizing the need for additional research. Overall, the findings of Study 1 point to the need for building awareness of individual vulnerability to misinformation. Future research should focus on how best to emphasize the threat of misinformation on a personal level while building psychological resistance.

In the second study, we found that the untrustworthiness of the source delivering the inoculation lowered participants' willingness to receive inoculations and that participants were less willing to take inoculations across political party lines. Participants were significantly less willing to watch videos from low- and medium-trust sources and partisan sources, while the high-trust source yielded results almost identical to those of the no-source condition. This indicates that anonymous or lesser-known organizations could potentially be effective providers of inoculation interventions.

Additionally, the findings underscore the importance of political affiliation in shaping individuals' attitudes towards misinformation inoculations. Participants tended to be more unwilling to engage in inoculations from sources that did not align with their political beliefs. However, even among party supporters, the likelihood of watching was lower than the no-source condition, showing that it is best to keep partisan politics and government far away from inoculation interventions. Any future campaigns for inoculation interventions should keep ‘the inoculator’ in mind when trying to reach the largest number of people. High-trust sources are more likely to garner acceptance, suggesting the importance of partnering only with reputable institutions to disseminate misinformation inoculations effectively.

Across both studies, we found that institutional trust was an important indicator of how likely participants would be to watch inoculation videos. As previously observed by Saltz *et al*. [48] institutional trust predicted support for misinformation interventions, in this case, inoculation videos. Even the least trustworthy source had more support from people who had higher trust in institutions, underscoring the importance of building up trust in institutions. This is a particularly concerning finding, especially considering that individuals with low levels of institutional trust are likely more susceptible to misinformation about those institutions. Consequently, they may be the demographic most in need of inoculation. This highlights the necessity to craft tailored appeals targeting individuals with low levels of institutional trust to encourage their engagement with interventions. Collectively, these findings present significant challenges, especially when targeting individuals with low trust in governments and researchers.

Roozenbeek *et al*.’s [27] study (that formulated the inoculation videos referred to in the present research) already attempted to circumvent the problem of a reluctant public by partnering with Google Jigsaw to run the videos as YouTube ads. This essentially put the videos in front of an audience without them having to actively choose to watch them. Interestingly, though the videos were developed in conjunction with Google Jigsaw [27], the word Google is found nowhere in the video. Instead, they state the source as ‘Truth Labs’, in conjunction with University of Cambridge, University of Bristol and Inoculation Science. Obscuring one of the sources and placing videos in front of people without their input are both possible solutions to the problems stated in this research, but they also bring up ethical concerns. Additionally, the results of our research imply deploying inoculations on a platform like YouTube and partnering with Google (even if not explicitly mentioned) may harm the trustworthiness provided by the academic institutions involved. While we didn’t test YouTube, Google or Cambridge, we did find that more people were more willing to watch an inoculation video from an academic institution (i.e. Harvard) than a tech company (i.e. Meta). Future research should look at what occurs when someone does take an inoculation from a source they don’t necessarily trust, or that they don’t recognize, as that could potentially affect the success of the inoculation. There is evidence that persuasion originating from low-trust sources is often rejected [39,43].

Governments, tech companies and researchers may roll out interventions in different ways such as embedding training videos on YouTube or other platforms—however, this poses entirely different challenges such as determining who gets the power to design and roll out these interventions and decide what is misinformation. This is a political and societal debate that goes beyond the scope of the paper. For the purpose of our study, we focus on whether people would engage with training voluntarily and find psychological features that may pose barriers to rolling out inoculation interventions.

We mostly focus on aspects of inoculation that could *prevent* engagement with inoculation interventions, but future research should look at possible features that could *encourage* engagement. Games and humorous videos are likely more appealing than more old-school text-based inoculations. However, the optimal strategies for the organic dissemination of such ‘fun’ interventions within social networks remain an open question. Additionally, a trustworthy source may be different for different groups, for instance, our research showed people planning to vote Republican tended to be less willing to watch inoculation videos from Harvard than people with other voting intentions. If inoculation interventions are to attract high voluntary engagement it seems necessary to use techniques like microtargeting or leveraging social media influencers to appeal to subgroups who may not naturally find inoculation appealing.

A major limitation of both studies is that they focused on self-reported perceptions and likelihoods. These were not compared to real-world actions. It is unclear if people who rated their own ability to detect misinformation highly are good at detecting misinformation or if they are being unrealistically optimistic. It may be useful in future research to have a baseline quiz to test participants' misinformation recognition skills to get a sense of whether the Dunning-Kruger effect [51] is at play. It is unknown if these participants had been presented with actual inoculation training videos from various sources, if they would be more or less likely to watch them than they reported. Future research on inoculation hesitancy should endeavour to give participants a choice to participate in real inoculation interventions to measure uptake more accurately.

Another notable limitation stems from using the likelihood of watching inoculation videos from Study 1 as a ‘no-source’ baseline condition. In Study 1, the survey introduces persuasion techniques associated with misinformation, attributing them to ‘Cambridge researchers.’ Although Cambridge is not explicitly restated in the subsequent questions, the survey continues to reference ‘researchers’ as the source, with the possible assumption likely being academic or university researchers. This shared characteristic with the Harvard University source in Study 2 introduces a potential confounding factor that necessitates careful consideration in interpreting the findings. Future research should explore the efficacy of truly ‘sourceless’ inoculations, as there is a possibility that such interventions might outperform those associated with low-trust sources.

It is also difficult to determine what other causes may be affecting the likelihood of engaging with inoculation interventions. Participants were not asked whether their perceived skill at detecting misinformation influenced their assertion of being unlikely to watch inoculation videos. The participants' belief in their higher detection skills and the idea that others would benefit more from the inoculation suggests a potential conviction in their immunity. However, it’s important to note that their reluctance to watch the videos may not necessarily be solely driven by this perceived immunity. In the second study, even the most trustworthy source inspired only a lukewarm likelihood of watching score of 4.52, the equivalent of halfway between ‘neither likely nor unlikely’ and ‘slightly likely’ on the Likert scale, showing a general lack of enthusiasm that goes beyond the source. Future research could explore this question in a more qualitative manner to more deeply investigate why the reluctance to engage with inoculation interventions exists.

## 7. Conclusion

### 7.1. Concluding remarks

Misinformation can be deeply harmful to democracy, health literacy and interpersonal relationships, so finding a viable psychological vaccine is an enormous step. However, it is only the first step in a much longer process of inoculating the public. Inoculation hesitancy is a significant obstacle. Lack of need and lack of trust operate as demotivational obstacles for people in assessing whether to engage with inoculation interventions. To gain voluntary participation in inoculation interventions, creating awareness of individual vulnerability, building trust in the inoculator and decentring partisan politics is essential. Campaigns should be tailored to address the unique concerns of different political affiliations and highlight the potential benefits of inoculation interventions, emphasizing that inoculation is important for everyone. Inoculation theory is worth scaling, but the road to achieving herd immunity against misinformation will be challenging. These findings are not meant to discourage the use of inoculation theory, but to offer insights into the obstacles ahead.

## Data Availability

The datasets and complete surveys are available on Open Science Framework: Study 1 [52] and Study 2 [53]. Electronic supplementary material is available online at [54].
